# Biocompatible nanocomposite cryogels with improved mechanical properties based on polyvinyl alcohol and carbon nanotubes for cardiovascular applications

**DOI:** 10.3389/fbioe.2025.1635837

**Published:** 2025-09-01

**Authors:** Maria A. Rezvova, Tatiana V. Glushkova, Kirill Yu Klyshnikov, Alexey L. Pykin, Tatiana B. Tkachenko, Tatiana N. Akentieva, Alexander E. Kostyunin, Pavel S. Onishchenko, Natalia N. Borisova, Marina P. Fokeeva, Vera G. Matveeva, Evgenia A. Senokosova, Evgeniya O. Krivkina, Evgeny A. Ovcharenko

**Affiliations:** ^1^ Research Institute for Complex Issues of Cardiovascular Diseases, Kemerovo, Russia; ^2^ Institute of Coal Chemistry and Material Science, Federal Research Centre of Coal and Coal Chemistry SB RAS, Kemerovo, Russia; ^3^ Institute of Fundamental Sciences, Kemerovo State University, Kemerovo, Russia

**Keywords:** polyvinyl alcohol cryogels, carbon nanotubes, biocompatible polymer materials, polymer heart valve prostheses, hemocompatibility, calcification

## Abstract

The unique properties of hydrogels have enabled their widespread use in various biomedical applications, including heart valve and blood vessel replacements. However, their current applications are limited by poor mechanical properties, including low strength, susceptibility to plastic deformation, and inadequate wear resistance, which are critical for load-bearing tissue replacements. Nanocomposite cryogels composed of polyvinyl alcohol and carbon nanotubes prepared using a DMSO/H_2_O solvent mixture, present a promising solution to address these limitations. Addition of nanoparticles up to 0.5% of the polymer mass showed a remarkable 59% increase in mechanical strength compared to single component cryogels. The reinforcement effect was also pronounced in strain hardening at large deformations. CNTs addition also enhanced the adhesion of Ea.hy 926 cells. However, the overall cell coverage on the cryogel surface was lower compared to control culture plastic, suggesting selective adhesion behavior. Comprehensive hemocompatibility testing showed minimal adsorption of protein molecules such as albumin and fibrinogen and no platelet adhesion when exposed to platelet rich plasma. Post contact platelet aggregation was same as untreated plasma. The studied materials also elicited minimal inflammatory response and no calcification unlike polytetrafluoroethylene which is clinically used, further proving biocompatibility. These findings suggest that PVA-based nanocomposite cryogels synthesized with dispersed CNTs hold significant promise for applications in cardiovascular surgery, particularly in the development of mechanically robust and biocompatible vascular grafts and heart valve prostheses.

## 1 Introduction

According to the World Health Organization (WHO), cardiovascular diseases (CVD) account for approximately 17.9 million deaths worldwide annually (World Health Organization, 2025). The prevalence of CVD continues to rise, and the affected age demographic is expanding. An increasing number of younger individuals now require drug therapy or surgical intervention to manage CVD. In many cases, the implantation of artificial substitutes, such as heart valve and vessel prostheses or cardiovascular patches, remains the only lifesaving option (Xue et al., 2023). However, the use of foreign materials in the body is associated with significant risks, including thrombosis, inflammatory reactions, and the subsequent need for repeat surgeries (Rezvova et al., 2023). These challenges highlight the significance of developing effective, safe, durable, and versatile solutions in the field.

Studies have shown that an ideal artificial implant should not only replicate the functions of native tissues but also meet specific biocompatibility requirements and mechanical properties (Tonti et al., 2021). In this context, synthetic hydrogels offer distinct advantages over clinically utilized elastomers such as polytetrafluoroethylene (PTFE) and polyethylene terephthalate (PET). Firstly, hydrogels, like other synthetic materials, can be universally applicable to patients and tailored for specific tasks through machine learning methods and computational modelling (Danilov et al., 2023). Moreover, hydrogels are three-dimensional, crosslinked macromolecular networks capable of retaining substantial amounts of water. This property imparts them with exceptional hydrophilicity, biocompatibility, and physical characteristics, such as a microstructure and elastic deformation properties that closely resemble certain biological tissues (Means and Grunlan, 2019). Owing to these unique characteristics, hydrogels have emerged as promising candidates for various biomedical applications, including heart valve and blood vessel substitution. However, their use remains limited by low mechanical properties, particularly in terms of tensile strength, stiffness and wear resistance, which undermines their effectiveness as replacements for load-bearing tissues. Current scientific efforts are focused on enhancing and optimizing the mechanical properties of hydrogels through methods such as forming interpenetrating networks (IPNs) (Zoratto and Matricardi, 2018), macromolecular crosslinking (Zafar et al., 2022), composite synthesis (Zhang et al., 2011), and dual crosslinking (Kim et al., 2020). Nonetheless, only a few innovative combinations of these approaches have successfully preserved high water content, akin to native tissues, while simultaneously achieving the requisite strength and stiffness (Hua et al., 2018).

Among the extensive range of known polymeric hydrogels, polyvinyl alcohol (PVA) stands out as particularly promising for cardiovascular applications. Its abundant hydroxyl groups enable the formation of three-dimensional crosslinked matrices through cryostructuring, a process involving the freezing and subsequent thawing of polymer solutions (Wang et al., 2021; Lozinsky et al., 2003). Unlike other hydrogel synthesis methods, such as chemical covalent bonding, polymerization, or radiation crosslinking, cryostructuring avoids the use of additional chemical agents, enabling the production of highly biocompatible and non-toxic materials. PVA hydrogels are chemically stable in water and biological fluids, resisting dissolution and hydrolysis while exhibiting limited swelling (Górska et al., 2021). However, the relatively low mechanical strength and wear resistance of PVA cryogels highlight the need for strategies to enhance these properties. One promising approach involves the integration of carbon nanotubes (CNTs) into the polymer matrix. The fabrication of cryostructured nanocomposites from aqueous PVA and CNT solutions typically requires prior nanoparticle functionalization (Tong et al., 2007; Pykin et al., 2025), as unmodified CNTs are hydrophobic and tend to form unstable dispersions in water (Singer et al., 2018). However, surface modification of nanoparticles with aggressive chemical agents can cause shortening of nanotubes and may lead to increased toxicity of the final material, which poses a serious concern for biomedical applications (Lavagna et al., 2022). In contrast, CNTs form stable dispersions in dimethyl sulfoxide (DMSO) and DMSO-water mixtures (Uddin et al., 2012; Mi et al., 2016). Notably, cryogels obtained using DMSO exhibit mechanical properties comparable to or, in some cases, superior to those of water-based gels, depending on the water ratio (Hyon et al., 1989).

In this study, we present the development of PVA-based nanocomposite cryogels reinforced with CNTs, using a DMSO-water solvent system, which has not been previously applied for the preparation of such nanocomposites. This solvent combination enables the formation of stable CNT dispersions without the need for aggressive chemical functionalization, thereby reducing the potential cytotoxicity of the resulting biomaterials. In addition to optimizing the synthesis conditions to obtain structurally uniform and stable cryogels, we provide a comprehensive characterization of their mechanical properties, including performance under cyclic loading–an essential factor for biomedical applications such as cardiovascular tissue replacement, where materials are subjected to continuous mechanical stress.

Importantly, comprehensive and reliable biocompatibility evaluation is crucial to ensure the safety of nanocomposite materials intended for biomedical use. However, such assessments are largely absent from previously published studies, which focus on basic mechanical properties (Tong et al., 2007; Pykin et al., 2025). In this work, we thoroughly evaluated the biocompatibility of the developed cryogels through a series of *in vitro* tests, including their interactions with cells, blood components, and proteins, as well as an *in vivo* study to assess the foreign body response. The results obtained underscore the high potential of the developed nanocomposite cryogels for biomedical applications.

## 2 Materials and methods

### 2.1 Fabrication of single component PVA cryogels

**FIGURE 1:**
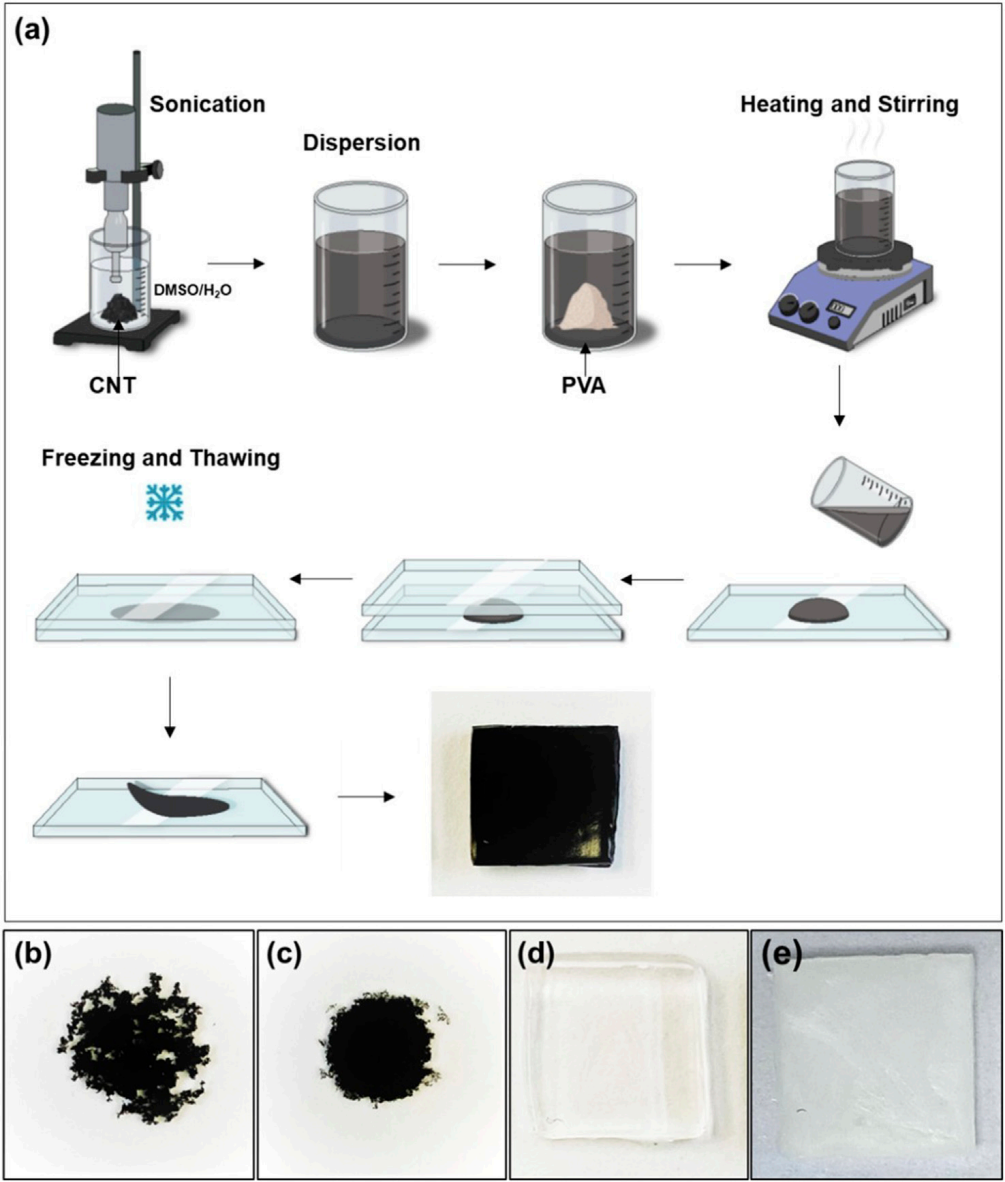
**(a)** Schematic representation of the preparation of PVA-CNT nanocomposite cryogels, **(b)** dried droplet of CNT dispersion in water, **(c)** dried droplet of CNT dispersion in DMSO/H_2_O mixture, **(d)** cryogel samples prepared without nanotubes in DMSO/H_2_O mixture and **(e)** in DI water.

Single component PVA cryogels were prepared *via* cryotropic gelation of polymer solutions in either deionized (DI) water (PVA-H_2_O) or a DMSO/H_2_O mixture (PVA-mix). The optimal concentration, temperature, number of freeze-thaw cycles, solvent mixture composition, and component ratio were previously determined based on preliminary tests of the samples’ mechanical properties. A PVA polymer sample with molecular weights ranging from 146,000 to 186,000 Da and a degree of hydrolysis of 99% was sourced from Sigma-Aldrich, United States. The polymer was dissolved either in a mixture of DI water (20% of the total volume) and DMSO or in pure DI water. The dissolution process was carried out at 95 °C for 2–3 h until a clear, homogeneous solution was obtained. Once cooled to room temperature, the solution was placed between two glass slides spaced 1 mm apart to ensure uniform sample thickness. This assembly then underwent a single freeze-thaw cycle, which involved cooling to −40 °C, gel structuring at −2 °C to −5 °C, and thawing at +8 °C. Following the cryotropic gelation process, the cryogels were washed in DI water for 48 h under continuous stirring to remove unlinked polymer residues and residual DMSO. The detailed parameters of the resulting samples are presented in Figure 1 and summarized in Table 1.

**TABLE 1:** Description of the obtained and studied polymer materials.

Materials	Description of the materials
Control Material	
ePTFE	Cardiovascular patch of the Gore-Tex brand (W.L. Gore and Associates, United States), thickness 0.6 mm. The material is used in clinical practice.
Cryogels based on polyvinyl alcohol (PVA)	
PVA-H_2_O	Cryogel based on PVA, obtained by a single freeze-thaw cycle of a 12 wt% polymer solution in water.
PVA-Mix	Cryogel based on PVA, obtained by a single freeze-thaw cycle of a 12wt% polymer solution in a DMSO/H_2_O mixture in a volume ratio of 80/20.
PVA-CNT	Cryogel based on PVA, obtained by a single freeze-thaw cycle of a 12wt% polymer solution in a DMSO/H_2_O mixture (80/20) with the addition of 0.5%^a^ carbon nanotubes.

^a^ Nanocomposite samples containing 0.1, 0.5, and 1 wt.% of nanotubes were tested in the uniaxial tensile test, while all other tests were conducted using the nanocomposite with 0.5 wt.% of nanotubes.

### 2.2 Preparation of nanocomposites based on PVA cryogels

CNTs exhibit poor dispersion in water due to their high surface hydrophobicity (Figure 1b). However, under sonication, these nanoparticles can form stable and homogeneous dispersions in both DMSO and DMSO/H_2_O mixtures (Figure 1c). For the synthesis of nanocomposites based on cryogels, single-walled carbon nanotubes with a diameter of 1.6 ± 0.4 nm and a length >5 µm (Sigma-Aldrich, United States) were dispersed in a DMSO/H_2_O mixture using an ultrasonic homogenizer Q500 (Qsonica, United States) for 15 min. The homogenizer (500 W maximum output) was operated at 20% amplitude, corresponding to an estimated output power of ∼100 W. A pre-weighed amount of dry PVA powder was then added to the resulting dark dispersion in various ratios (100:1, 100:0.5, and 100:0.1 relative to the weight of CNTs). The mixture was heated with stirring until a uniform suspension was formed. The nanocomposites were subsequently cryostructured using the same protocol as described for single component cryogels (see Section 2.1). The scheme for cryogel synthesis and the characteristics of the samples produced are presented in Figure 1 and detailed in Table 1. Nanocomposite samples containing 0.1, 0.5, and 1 wt.% of nanotubes were tested in the uniaxial tensile test, while all other tests were conducted using the nanocomposite with 0.5 wt.% of nanotubes, referred to as PVA-CNT.

### 2.3 Structure and characterization of cryogel nanocomposites

#### 2.3.1 Infrared spectroscopy (ATR-FTIR)

The infrared spectra of the analyzed film samples were obtained using an “Infralum FT-08”IR spectrometer (Lumex, Russia). Spectra were recorded with a Multiple-Bounce Attenuated Total Reflectance (MBATR) accessory equipped with a ZnSe prism (Pike, United States). Each lyophilized sample was securely attached to the surface of a flat crystalline plate using a press apparatus. Spectral data were collected in the range of 4,000–500 cm^−1^, with a resolution of 4 cm^−1^ and an accumulation of 256 scans.

#### 2.3.2 Thermogravimetric analysis (TGA)

The thermal stability of the obtained nanocomposite PVA cryogels was evaluated using thermogravimetric analysis (TGA) on a STA 449 F3 Jupiter^®^ thermal analyzer (NETZSCH Geratebau GmbH, Germany). Lyophilized cryogel samples weighing 3 mg were heated in a platinum crucible from 25 °C to 600 °C under a nitrogen atmosphere at a heating rate of 5 °C/min.

#### 2.3.3 Scanning electron microscopy (SEM)

The internal structure and surface morphology of the fabricated polymeric samples were analyzed using scanning electron microscopy (SEM). Before imaging, the cryogels were dried using a FREEZONE 2.5 tabletop vacuum lyophilizer (LABCONCO, United States) at −40 °C under a pressure of <0.133 mbar. For internal structure analysis, the dried samples were fractured in liquid nitrogen to expose their cross-sections. The samples were then mounted on SEM stubs and sputter-coated with a gold-palladium layer using an Emitech SC7640 vacuum coater (Quorum Technologies, England) to enhance conductivity. Imaging was performed with an S-3400N scanning electron microscope (Hitachi, Japan) under high vacuum at an accelerating voltage of 10 kV in secondary electron mode.

#### 2.3.4 Assessment of water content

To evaluate the water content of the obtained cryogels, wet samples of about 1 × 1 cm^2^ (n = 10) were weighed and dried for 48 h under thermostatic conditions at a temperature of 37 °C. After which, the weight of the dry polymers was assessed and the water content for each type of sample was calculated according to the equation below:

$$water\;content=\frac{cryogel\;initial\;weight-cryogel\;dry\;weight}{cryogel\;initial\;weight}*100\%$$

#### 2.3.5 Contact angle assessment

The water contact angle was determined using the “sessile drop” method on experimental equipment. A 15 µL droplet of distilled water was placed onto the surface of the samples and stabilized for 5 s for each measurement. All tests were conducted at room temperature. The obtained images were processed using the Contact Angle plugin in the ImageJ software program (National Institutes of Health, Bethesda, United States). The analysis was repeated 10 times for each sample group.

### 2.4 Mechanical testing of cryogel nanocomposites

#### 2.4.1 Uniaxial tension

Mechanical properties of the obtained materials were evaluated under uniaxial tension in accordance with ISO 37:2017 at 37 °C. Test samples were prepared using a ZCP 020 cutting press (Zwick GmbH and Co. KG, Germany) and a specially shaped knife (B083, as specified in ISO 37:2017), with *n* = 7–8 samples. Material directionality was not considered due to the isotropic nature of the samples.

Mechanical testing was conducted on a “Z-series” universal testing machine (Zwick GmbH and Co. KG, Germany) equipped with a force sensor of 50 N. The crosshead speed was set to 50 mm/min. The Ultimate Tensile Strength (UTS, MPa) was defined as the maximum load divided by the initial cross-sectional area of the sample. Deformation properties were assessed by the relative elongation at break (corrected for the mode of sample failure, %), and Young’s modulus (MPa) was calculated within the range of small deformations (0.4–0.6 MPa). Sample thickness was measured using a thickness gauge with a permissible error of ±0.01 mm and a clamping force not exceeding 1.5 N.

#### 2.4.2 Elastic hysteresis

Elastic hysteresis was investigated using equipment and conditions analogous to those employed in uniaxial tensile testing of specimens. During the hysteresis evaluation, the loading rate (mm/min) was set at half the target elongation value for the following strain levels: 10%, 20%, 30%, 40%, 50%, 75%, 100%, 150%, and 200%. For each step, eight loading-unloading cycles were performed, with the first five serving as stabilization cycles and the last three considered valid for determining the working stress at a given elongation (Diani et al., 2009). The cryogel specimens were maintained in a hydrated state throughout the experiment. When changing the elongation step, the specimens were allowed to relax for 1 minute.

Elastic hysteresis results were assessed using a program written in Python 3.10, which incorporated functionalities for calculating irreversible deformation and peak stress loss Test data–presented as “elongation (%)” and “stress (MPa)” for each loading-unloading cycle–were used to determine the loading start point and the peak point (unloading start). Based on these parameters irreversible deformation, peak stress drop, and Young’s modulus (calculated as the secant modulus between 0 MPa and UTS) were computed.

### 2.5 Cytotoxicity of cryogel nanocomposites

The Ea.hy926 cell line was selected as the experimental endothelial cell model. This hybridoma cell line is derived from human endothelial cells and A549/8 cells. Cells were cultured in DMEM/F12 nutrient medium (11320033, Thermo Fisher Scientific, United States) supplemented with HAT (H0262, Sigma Aldrich, United States), 10% fetal bovine serum (26140079, Thermo Fisher Scientific, United States), antibiotics (0378016, Thermo Fisher Scientific, United States), and amphotericin B (15290018, Thermo Fisher Scientific, United States). Subculturing was performed when the culture reached 70% confluency, and cells were detached using a 0.025% trypsin-EDTA solution (15400054, Thermo Fisher Scientific, United States). All experiments were carried out under sterile conditions, with cells maintained in a CO_2_ incubator at 5% CO_2_ and high humidity. Ea.hy926 cells were seeded onto fixed matrix samples at a density of 50,000 cells per well and cultured in complete nutrient medium for 3 days, with media changes on days 1 and 3. The control group consisted of samples in a 24-well plate without matrices, seeded with an equivalent number of cells and cultured under identical conditions. The surface area of each tested sample was approximately 113 mm^2^.

After 3 days, cell adhesion and viability were assessed using fluorescence microscopy (n = 4, for each material type), metabolic activity was measured using colorimetric methods (n = 6), and proliferative activity was evaluated *via* confocal fluorescence microscopy (n = 2).

#### 2.5.1 Cell viability

Cells were stained with Hoechst 33342 nuclear dye (10 μg/mL, 14533, Sigma Aldrich, United States) for 10 min and ethidium bromide (30 μg/mL, 46067, Sigma Aldrich, United States) for 1 min. Cell counts on both sample surfaces and culture plastic were performed using an inverted microscope, Axio Observer Z1 (Carl Zeiss, Oberkochen, Germany), with five random fields of view analyzed for each replicate. For Hoechst 33342, fluorescence was observed at an excitation/emission wavelength of 350/461 nm, while an appropriate wavelength filter was used for ethidium bromide.

The cell count from the field of view was recalculated to an area of S = 1 mm^2^. The relative number of dead cells was calculated using the formula: absolute number of dead cells*100%/absolute number of all adhered cells. The relative number of living cells was determined by subtracting the percentage of dead cells from 100% of the adhered cell population.

#### 2.5.2 Cell proliferative activity

Cell proliferative activity was evaluated using the Click-iT™ Plus EdU Cell Proliferation Kit for Imaging (C10637, Thermo Fisher Scientific, United States). Cells were incubated with the EdU reagent for 16 h and subsequently stained according to the manufacturer’s protocol. Following the staining procedure, cells were further stained with DAPI nuclear dye (10 μg/mL, D9542, Sigma Aldrich, United States) for 30 min. Preparations were analyzed using the LSM700 scanning confocal microscope (Carl Zeiss, Germany). Ten randomly selected fields of view were examined for each sample at ×200 magnification, with two samples analyzed for each polymer type. Quantitative image analysis was performed using ImageJ software (National Institutes of Health, Bethesda, MD, United States). The analysis included counting the total number of cells and the number of proliferating cells within each field of view. The relative number of proliferating cells was calculated using the following formula: number of proliferating cells in the field of view *100/total number of cells in the field of view.

#### 2.5.3 Cell metabolic activity

Cell metabolic activity was evaluated using a colorimetric method with the Cell Cytotoxicity Assay Kit - Colorimetric (ab112118, Abcam, Cambridge, United Kingdom). The reagent, diluted to its working concentration (1:5 with the nutrient medium), was added to the wells containing the samples and incubated for 3 h at 37 °C. Following incubation, 200 µL of the reagent from each well containing samples was transferred to a 96-well plate. Optical density was measured at two wavelengths, 570 nm and 605 nm, using the Multiskan Sky spectrophotometer (Thermo Fisher Scientific, United States).

### 2.6 Hemocompatibility assessment of cryogel nanocomposites *in vitro*

#### 2.6.1 Protein adhesion

To assess hemocompatibility–one of the critical parameters for materials intended for blood-contacting applications–the adsorption of albumin and fibrinogen proteins on the surface of the nanocomposite materials was analysed. Polymer samples were immersed in solutions containing the respective proteins. The amount of protein desorbed from the sample surfaces and released into the SDS solution was quantified using the Micro BCA™ Protein Assay Kit (abcam, Cambridge, United Kingdom). Protein concentrations were measured spectrophotometrically at 562 nm.

#### 2.6.2 Hemolysis

Experiments involving donor blood samples were conducted in accordance with the Declaration of Helsinki and were approved by the Local Ethics Committee (LEC) of the Research Institute for Complex Issues of Cardiovascular Diseases, Kemerovo. The study’s compliance is documented in LEC meeting protocol No. 08/1, dated 27 August 2021.

The hemocompatibility of the nanocomposites was evaluated in accordance with the key requirements of the ISO 10993-4:2017 standard. Specifically, the degree of hemolysis and platelet adhesion were examined after the interaction of the polymer matrices with donor blood anticoagulated with citrate. The degree of hemolysis was determined spectroscopically by quantifying the hemoglobin released from erythrocytes following contact with the test materials. The optical density (OD) of the negative control (a physiological solution mixed with citrated blood) represented the complete absence of hemolysis, while the OD value from distilled water mixed with citrated blood corresponded to 100% hemolysis.

#### 2.6.3 Platelet adhesion

To evaluate platelet adhesion and the transformation level of adhered platelets, polymer material samples were incubated in platelet-rich plasma (PRP). The PRP was derived from freshly collected citrated donor blood by centrifugation at 1,200 rpm for 10 min. Following incubation, the samples were carefully washed, fixed in a 2% glutaraldehyde solution, and dehydrated through a graded series of ethanol concentrations. A conductive Au/Pd coating was subsequently applied to the sample surfaces using the EM ACE200 vacuum system (Leica Mikrosysteme GmbH, Austria). The surface morphology of the polymer materials, both before and after platelet interaction, was analyzed using an S-3400N scanning electron microscope (Hitachi, Japan) under high-vacuum conditions with an accelerating voltage of 15 kV. For comparative assessment of hemocompatibility, ePTFE was included in the study.

#### 2.6.4 Platelet aggregation

To evaluate platelet aggregation, the cryogel samples were exposed to PRP at 37 °C for 5 min, with intact PRP serving as the control group (n = 6). Then adenosine 5′-diphosphate (ADP)-induced platelet aggregation were assessed. Induced aggregation was triggered using 4 μm/L ADP (030, Tehnologia-standart, Russia), and measurements were performed using the APACT 4004 platelet analyzer (LABiTec, Germany).

### 2.7 Biocompatibility assessment of cryogel nanocomposites *in vivo*

Animal experiments were conducted following the ARRIVE 2.0 guidelines and were approved by the Local Ethics Committee (LEC) of the Research Institute for Complex Issues of Cardiovascular Diseases, Kemerovo. The study’s compliance is documented in the extract from the LEC meeting protocol No. 08/1, dated 27 August 2021.

*In vivo* tests were conducted on male Wistar rats (90–100 g, n = 5 per group). All surgical procedures were performed under isoflurane inhalation anesthesia. Biomaterial samples (0.6 × 0.6 cm^2^) were sterilized with 70% ethanol and thoroughly rinsed with sterile saline solution. The fur on the dorsal region of each rat was carefully shaved, and the implantation site was disinfected with a skin antiseptic. Under aseptic conditions, six subcutaneous pockets were created per rat through individual 0.5 mm incisions along the spine–three on the right side and three on the left. The prepared samples were systematically inserted into these pockets. The incisions were then closed using non-absorbable polyester sutures (Lavsan 4.0, Lintex, Russia).

To assess tissue response to the implanted samples, extractions were performed at 14- and 60-day post-implantation. During extraction, the implants, the encapsulating layer, and approximately 5 mm of adjacent unmodified soft tissue were excised. The samples were rinsed in 0.9% NaCl solution and preserved in Neg-50 rapid tissue freezing medium (Cat. No. 6502, Thermo Fisher Scientific). Using a microtome-cryostat HM525 (Thermo Fisher Scientific), 6 μm-thick cross-sectional slices were prepared and mounted on slides for further analysis.

#### 2.7.1 Histology

Structural changes in the polymer materials and their integration with surrounding tissues were evaluated using histological techniques. For this purpose, the sections were first fixed in 4% paraformaldehyde for 10 min, then washed three times (5 min per wash) in distilled water using a shaker (Polymax 1040, Heidolph) set at 30 rpm.

The sections were subsequently stained with hematoxylin and eosin (H&E) (BioVitrum, Russia) and Alizarin Red S (Reachem, Russia) to detect calcium ions, following the respective manufacturers’ protocols. After staining, the sections were mounted under coverslips using Vitrogel mounting medium (BioVitrum).

#### 2.7.2 Immunohistochemistry

The intensity of the inflammatory response following the implantation of the investigated polymer membranes was evaluated using immunohistochemical staining with antibodies targeting the pan-leukocyte marker CD45 (ab10558, Abcam) and the α-SMA marker (ab240678, Abcam). Prior to staining, sections were fixed in 4% paraformaldehyde at room temperature for 10 min and then washed three times (5 min per wash) in phosphate-buffered saline (PBS, pH 7.4) on a shaker. The immunohistochemical procedure was performed using the NovoLink Polymer Detection System kit (Leica Microsystems Inc.) according to a modified manufacturer’s protocol. To quench endogenous peroxidase activity, sections were treated with a 4% hydrogen peroxide solution (Peroxidase Block) for 5 min, rinsed twice in PBS, and then blocked to prevent nonspecific antibody binding by incubation with a 0.4% casein saline solution supplemented with auxiliary reagents (Protein Block) for 1 hour. Primary antibodies were diluted according to the manufacturer’s instructions in a 1% bovine serum albumin saline solution at a 1:1,000 ratio. The sections were incubated with primary antibodies for 20 h inside a sealed container at +4 °C, followed by three PBS washes and a 30-min incubation with secondary anti-rabbit antibodies (Novolink Polymer). After three additional washes in PBS, the sections were treated with a 0.087% diaminobenzidine solution for 2 min, rinsed in distilled water for 5 min, and counterstained with hematoxylin (from the provided kit) for 10 min. The sections were then blued in running tap water for 5 min, sequentially dehydrated in three changes of 95% ethanol (5 min each), and cleared in three changes of xylene (5 min each). Finally, they were mounted under coverslips using Vitrogel mounting medium (BioVitrum).

Histological sections stained with anti-CD45 and α-SMA antibodies were scanned using an automated laboratory microscope (MT5300L, Meiji Techno, Japan), and the resulting slides were analyzed using QuPath software (v.0.6.0). Quantification of CD45^+^ cells was performed in the peri-implant connective tissue capsule, specifically in areas located within 200 µm of the polymer sample surfaces. For each material type, CD45^+^ cells were counted in 20 randomly selected fields of view (200 × 200 µm).

#### 2.7.3 Calcium quantification

Calcium content in the explanted samples (n = 5 for each group) was quantified using a spectrophotometric method. First, the biomaterial samples, along with their fibrous capsules, were lyophilized for 24 h, after which their weight was recorded. The samples were then subjected to hydrolysis in hydrochloric acid until they were completely dissolved. The calcium content in the resultant solution was determined using the Multiskan Sky spectrophotometer (Thermo Fisher Scientific) at a wavelength of 575 nm, employing the Calcium Assay Kit (ab102505, Abcam).

### 2.8 Statistical analysis

Statistical analysis was performed using GraphPad Prism 7.0 (GraphPad Software, San Diego, CA, United States). The normality of distribution was assessed using the Kolmogorov-Smirnov test. The statistical significance of differences between groups was determined by analysis of variance (ANOVA) with Fisher’s parametric test for *post hoc* comparisons. For nonparametric data, differences between groups were evaluated using the Kruskal–Wallis test with Dunn’s correction for multiple comparisons. When the distribution was normal, results are presented as the mean ± standard deviation (SD); for non-normal distributions, data are expressed as the median with 25th and 75th percentiles (Me [25%; 75%]). A p-value of <0.05 was considered statistically significant.

## 3 Results and discussion

### 3.1 Fabrication of nanocomposites based on PVA cryogels

Introducing CNTs into the polymer matrix is a well-established approach for enhancing the mechanical properties of materials intended for diverse applications (Abubakre et al., 2023). With the evolution of automation technologies and advancements in medicine, there has been a rising interest in smart hydrogel nanocomposite materials. These are targeted for applications such as electronic skin, personal health monitoring, humanoid robotics, cardiovascular substitutes, and more (Huang et al., 2022). Beyond their exceptional mechanical properties, the efficacy of hydrogels integrated with carbon nanomaterials in these domains can be attributed to their elevated electrical conductivity and established biocompatibility (Ye et al., 2021; Van den Broeck et al., 2019). Electrical conductivity is critical for developing hydrogels tailored to myocardial patches or *in vitro* cell culture platforms (Sauvage et al., 2024). For applications such as heart valves or vascular grafts, which we have identified as our target applications, hydrogels must provide adequate mechanical strength, resistance to cyclic loading, and long-term durability, while maintaining biocompatibility, particularly hemocompatibility and resistance to calcification (Rezvova et al., 2023).

PVA cryogels occupy a unique position among medical hydrogels due to their distinctive formation mechanism, which involves the cryostructuring of polymer solutions to create a three-dimensionally crosslinked network (Lozinsky et al., 2003). In such cryotropic gels, crosslinks are established through physical interactions (Bercea, 2024). When fabricating hydrogel nanocomposites *via* cryostructuring, one of the key challenges is achieving uniform CNT distribution in hydrophilic solvents. This difficulty arises from the hydrophobic nature of the CNT surface, which promotes nanotube aggregation or agglomeration in water solutions (Tong et al., 2007). Various strategies have been explored to mitigate this issue, including nanotube oxidation and alternative methods to enhance their hydrophilicity (Tong et al., 2007; Pykin et al., 2025; Huang et al., 2011). However, these approaches have significant drawbacks: the use of aggressive chemicals can degrade or shorten nanotubes, increase toxicity, and compromise the mechanical strength of the resulting nanocomposites (Lavagna et al., 2022). To achieve a uniform dispersion of CNTs within the PVA polymer matrix, we used a mixture of DMSO and water as the solvent. This combination enabled the formation of stable and uniform nanoparticle dispersions under ultrasonic treatment (Figure 1c). The resulting cryogels were opaque and black, in contrast to the single component cryogel, which was homogeneous, colourless, and completely transparent (Figure 1e). As reference samples, water-based cryogels exhibited a characteristic macroscopic structural pattern, attributed to water crystallization processes (Figure 1e).

The formation of three-dimensional crosslinked structures in PVA solutions follows a general principle based on the development of a bicontinuous system, consisting of a concentrated (polymer-rich) phase and a diluted (polymer-poor) phase. This process brings PVA chains into proximity, facilitating the formation of crystalline domains (crystallites) and intermolecular hydrogen bonds (Holloway et al., 2013). In aqueous PVA solutions, cryostructuring is primarily driven by the formation of ice crystals during cooling, which induces phase separation (Holloway et al., 2013). However, when a DMSO/H_2_O mixture is used as the solvent, crystallization does not occur, except at very low temperatures. Instead, rapid cooling reduces polymer solubility, leading to localized polymer concentration and subsequent gelation due to crystallite formation, even in a homogeneous solution. The transparency of cryogels prepared in the DMSO/H_2_O system, in contrast to the opacity of water-based cryogels, can be primarily attributed to the absence of phase separation and the formation of a more homogeneous polymer network, which reduces light scattering (Takeshita et al., 2002). The rapid gelation observed in dimethyl sulfoxide-based PVA solutions is associated with the strong interactions between DMSO and H_2_O molecules, which exceed those between the polymer and solvent molecules. These interactions displace the solvation layer around the polymer chains, allowing hydroxyl functional groups on the polymer backbone to establish hydrogen bonds with one another (Wu et al., 2021). Furthermore, hydrogen bond formation between polymer molecules also occurs during the washing stage of DMSO/H_2_O cryogels in water. This process effectively replaces DMSO, a thermodynamically good solvent for PVA, with water, a poor solvent, further reinforcing intermolecular interactions.

### 3.2 Structure and characterization of cryogel nanocomposites

#### 3.2.1 ATR-FTIR

**FIGURE 2:**
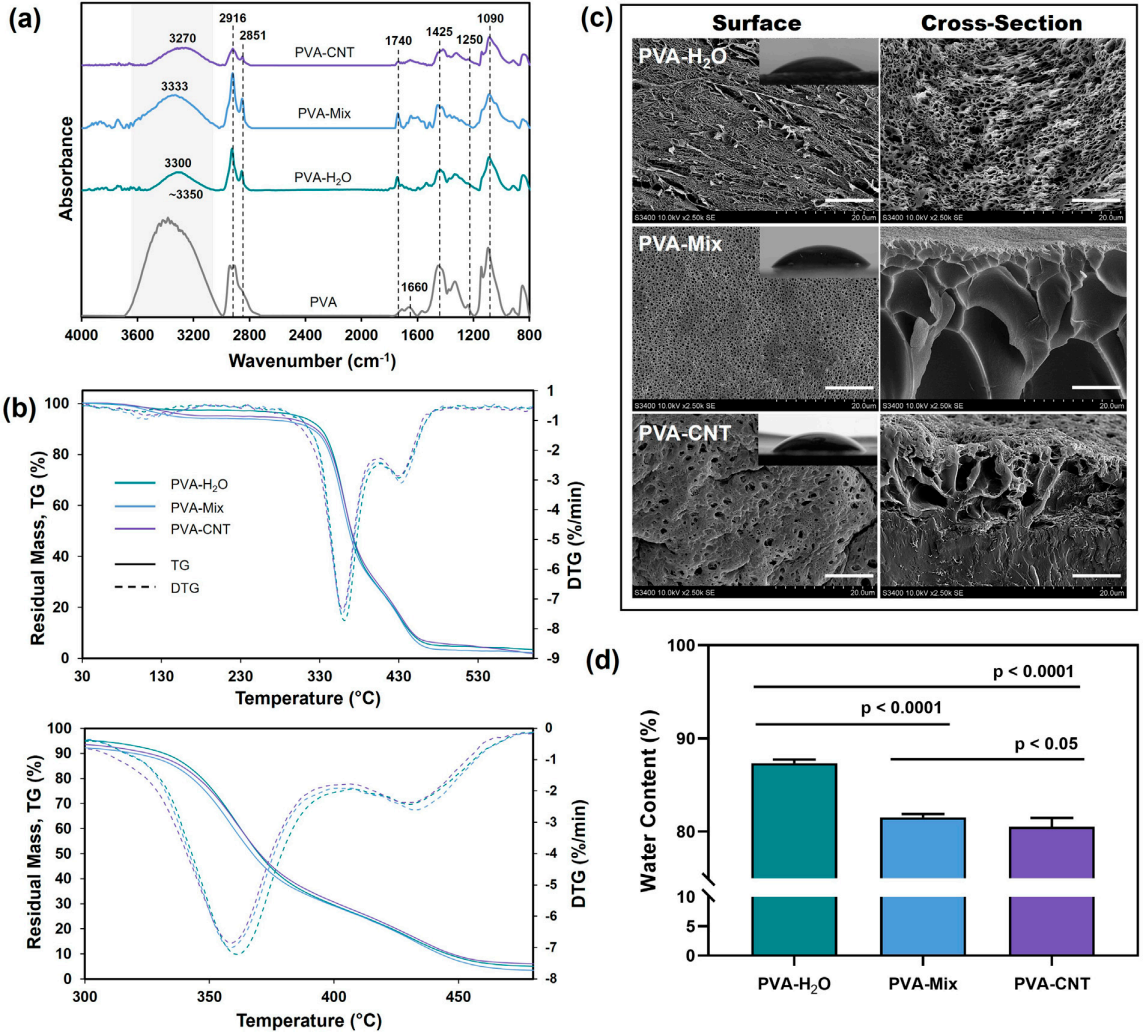
Structure and characterization of cryogel nanocomposites. **(a)** ATR-FTIR, **(b)** TGA, **(c)** SEM images of the surface and fracture surface morphologies of cryogels (scale bars: 10 μm), and mages obtained from water contact angle measurements and **(d)** water content of cryogels. Statistical significance of differences between groups was determined by analysis of variance (ANOVA) with Fisher’s parametric test for *post hoc* comparisons. Data are presented as mean ± SD (n = 10).

To determine the differences in the structure and properties of single component PVA cryogels and nanocomposites, and to evaluate the influence of DMSO on cryogel formation, ATR-FTIR spectra were analysed for the following samples: PVA-H_2_O, PVA-Mix, PVA-CNT, and commercial linear PVA. In the spectra of all studied samples, characteristic absorption bands corresponding to the valence vibrations of O-H inter- and intramolecular hydrogen bonds were observed in the range of 3,350–3,270 cm^−1^ (Figure 2a) (Sa’adon et al., 2021). A shift of this broad band towards the spectral region of 3,600–3,500 cm^−1^ was noted for linear PVA, which may be attributed to the presence of a greater number of free OH groups compared to cryogels. Two peaks at 2,916 and 2,851 cm^−1^ were attributed to the valence vibrations of CH_2_ and CH groups in the aliphatic carbon chain of PVA. In the case of nanocomposites, these peaks also correspond to the C-H bonds present in the graphene structure (Figure 2a) (Korbag and Mohamed Saleh, 2016; Chaturvedi et al., 2014). Characteristic absorption bands of the valence vibrations of the C=O bond in the ester group were detected at 1,740 cm^−1^, while asymmetric stretching of the carbonyl groups (C=O) in PVA was observed at 1,660 cm^−1^. Additionally, valence vibrations of C-C bonds (1,145 cm^−1^) and C-O bonds of secondary alcohols (1,090 cm^−1^) were observed. The band at 1,425 cm^−1^ was attributed to the deformation vibrations of the C-H bonds (Figure 2a) (Dostert et al., 2016). The overall decrease in the absorption spectrum intensity of nanocomposites compared to the original cryogels may be related to the scattering effect of reflected energy on CNTs.

#### 3.2.2 TGA

The cryogel materials studied (PVA-H_2_O, PVA-Mix, PVA-CNT) exhibited three distinct stages of thermal decomposition, which were clearly identified through TGA and DTG curves (Figure 2b). The thermal decomposition of PVA cryogels is a complex process that results in the formation of decomposition products such as water, acetaldehyde, alcohols, acetone, and carboxylic acids in various ratios depending on the experimental conditions. The first stage commenced at approximately 80 °C and reached its maximum decomposition rate at around 110 °C–120 °C. This stage is attributed to the presence of residual water within the cryogel network, as well as the evaporation of acetaldehyde (Fathi et al., 2011). The second stage of thermal degradation corresponded to the melting process of the polymer network and the partial dehydration of the polymer chains, leading to the formation of polyenes, occurring within the temperature range of 330 °C–380 °C. The third stage began at approximately 420 °C and was associated with the decomposition of polyenes and the breakdown of macro radicals, ultimately resulting in the destruction of the main hydrocarbon chain (Wang et al., 2019).

The overall thermogravimetric profile was consistent with previously reported data for other types of PVA hydrogels, with some variations attributed to differences in the polymer’s molecular weight, molecular weight distribution, and synthesis conditions (Crețu et al., 2022).

From the mentioned thermograms, thermogravimetric parameters were determined for the two decomposition stages characterized by the highest weight loss. These parameters include the onset temperature (T_onset_), the temperature of maximum decomposition rate (T_max_), and the temperature marking the end of each stage (T_end_), along with the residual mass (Residual mass). The values of these parameters are presented in Table 2.

**TABLE 2:** Thermogravimetric parameters of the studied cryogels: onset temperature of decomposition (T_onset_), temperature of maximum decomposition rate (T_max_), temperature corresponding to the end of each stage (T_end_), and residual mass (Residual mass).

Sample name	2 days stage of decomposition			3 days stage of decomposition			Residual mass, %
	T_onset_, °C	T_max_, °C	T_end_, °C	T_onset_, °C	T_max_, °C	T_end_, °C	
PVA-H_2_O	329.4	354.5	374.2	420.2	432.2	453.4	2.83
PVA-Mix	335.8	357.2	375.2	419.8	433.4	453.3	2.29
PVA-CNT	337.5	360.0	377.0	419.6	428.5	452.0	1.91

During the first stage of thermal decomposition, the cryogels lost residual water that was not bound to the polymer matrix, with no significant differences observed in the nature of the process across samples. The second stage of decomposition, associated with dehydration and the melting of polymer molecules (Table 1), presented the most intriguing differences in interpretation. Aqueous cryogels exhibited lower thermal stability compared to cryogels prepared using the DMSO/H_2_O mixture, likely due to differences in structural organization. As previously noted, aqueous cryogels form through the crystallization of ice and microphase separation, whereas cryogels prepared from the DMSO/H_2_O mixture are more homogeneous and contain a higher number of crystallites. The increased number of crystallites and hydrogen bonds between macromolecules in the DMSO/H_2_O cryogels likely required additional energy for disruption, resulting in higher temperatures for melting and dehydration (Table 1).

Thermogravimetric analysis results indicated that the incorporation of carbon nanoparticles enhanced the thermal resistance of PVA cryogels, as evidenced by a reduced decomposition rate compared to pure PVA (Figure 2b; Table 1). The shift in T_max_ to higher temperatures suggests a delayed decomposition of polymer molecules in contact with thermally stable carbon nanotubes (Corcione and Frigione, 2012). Additionally, the improved thermal stability of the nanocomposites can be attributed to the enhanced thermal conductivity of CNTs, which likely facilitated heat dissipation within the composite structure (Huxtable et al., 2003).

#### 3.2.3 SEM

SEM images of the synthesized PVA-based cryogels (Figure 2c) (Crețu et al., 2022) reveal a three-dimensional porous network with interconnected pores. Surface and cross-sectional views highlight structural differences influenced by the solvent type and the presence of a nanofiller. In aqueous cryogels, pores formed by ice crystal melting measure approximately 1–2 µm in diameter, with cavity shapes reflecting the morphology of ice crystals and exhibiting a regular pattern. Additionally, elongated pores with smaller diameters are observed on the surface. Cryogels synthesized using a DMSO/H_2_O mixture exhibit circular surface pores (∼200 nm in diameter), while the bulk structure contains cavities of varying shapes and sizes, reaching 10–15 µm. At higher magnifications, smaller pores are also visible on the cavity walls.

Within the polymer matrix, CNTs formed evenly distributed fibrous elements with enhanced electron density (Figure 2c). The introduction of nanotubes altered the nanocomposite surface structure compared to pure cryogels, leading to the formation of larger pores and distortion of cross-sectional cavities. The porous structure of PVA cryogels provides a large contact surface with water, high hydrophilicity, and consequently, enhanced biocompatibility (Li et al., 2020). These pores can also serve as encapsulation sites for bioactive compounds (Lozinsky et al., 2003) and contribute to the elasticity of the polymer matrix (Wang et al., 2021). Depending on the application, pore size plays a crucial role. For materials intended for blood-contact applications, smaller pores are generally more desirable. Several studies have demonstrated that materials with larger pores are often associated with increased vascularization and fibrosis, as well as allowing for deeper calcification during subcutaneous implantation (Hernandez and Woodrow, 2022).

#### 3.2.4 Water content

Due to the large number of hydroxyl groups in their structure, PVA cryogels can swell in aqueous solutions and retain a significant amount of water, exceeding the mass of the polymer matrix by several times (Wang et al., 2021). At the same time, the physical three-dimensional crosslinks formed *via* cryostructuring constrain the shape and volume of the hydrogels (Razavi et al., 2019). The water content can vary depending on the synthesis conditions of the polymer cryogel, including the polymer concentration in the initial solution, the number of freeze-thaw cycles, the freezing temperature and rate, the type of solvent, and other factors.

Our investigation of the water content in the obtained cryogels showed a significant decrease when water was replaced with a DMSO/H_2_O mixture, dropping from 87.35% to 81.57% (p < 0.05) (Figure 2d). This decrease is attributed to the looser structure of hydrogels prepared with pure water, which contain fewer crosslinks. The introduction of CNTs into the nanocomposite led to a further decrease in water content by approximately 1.5% (p < 0.05). Overall, the challenge of maintaining high water content while improving mechanical properties is particularly relevant for the development of synthetic medical cryogels, with values above 70% considered high (Means and Grunlan, 2019).

#### 3.2.5 Contact angle assessment

The PVA-H_2_O cryogels, prepared from aqueous polymer solutions, showed the lowest contact angle of 40.6° ± 4.8° (Figure 2c), reflecting the highest surface hydrophilicity. While PVA-mix and PVA-CNT cryogels showed slightly higher water contact angles of 46.3° ± 3.7° and 45.6° ± 5.9°, respectively, compared to PVA-H_2_O, the difference was not statistically significant according to one-way ANOVA with Dunn’s correction test (p > 0.05). Nevertheless, since the PVA-mix cryogels showed a contact angle comparable to that of PVA-CNT, this may be attributed to the influence of DMSO on the surface organization and polymer-solvent interactions, which correlates with the SEM data. Additionally, it is worth noting that the water content decreases more substantially when transitioning from pure water as a solvent to a water/DMSO mixture (section 3.2.4), which likely contributed to the reduced surface wettability of the polymer cryogels.

Therefore, it can be suggested that the incorporation of carbon nanotubes does not significantly influence surface wettability. Overall, all samples remain within the range typical for moderately hydrophilic materials.

### 3.3 Mechanical testing of cryogel nanocomposites

Despite the high hydrophilicity and unique biocompatibility of PVA cryogels, their use in prosthetic heart valve leaflets is limited due to their low mechanical strength, which is inferior to that of biological tissues and certain elastic synthetic polymers (Ovcharenko et al., 2017; Li et al., 2019). It has been suggested that swelling of the crosslinked network by the solvent significantly reduces viscosity caused by friction between polymer chains, meaning that a high-water content leads to a decline in the gel’s mechanical properties (Gupta et al., 2011). However, reinforcing the polymer matrix while maintaining its water content remains a significant challenge.

#### 3.3.1 Uniaxial tension

**FIGURE 3:**
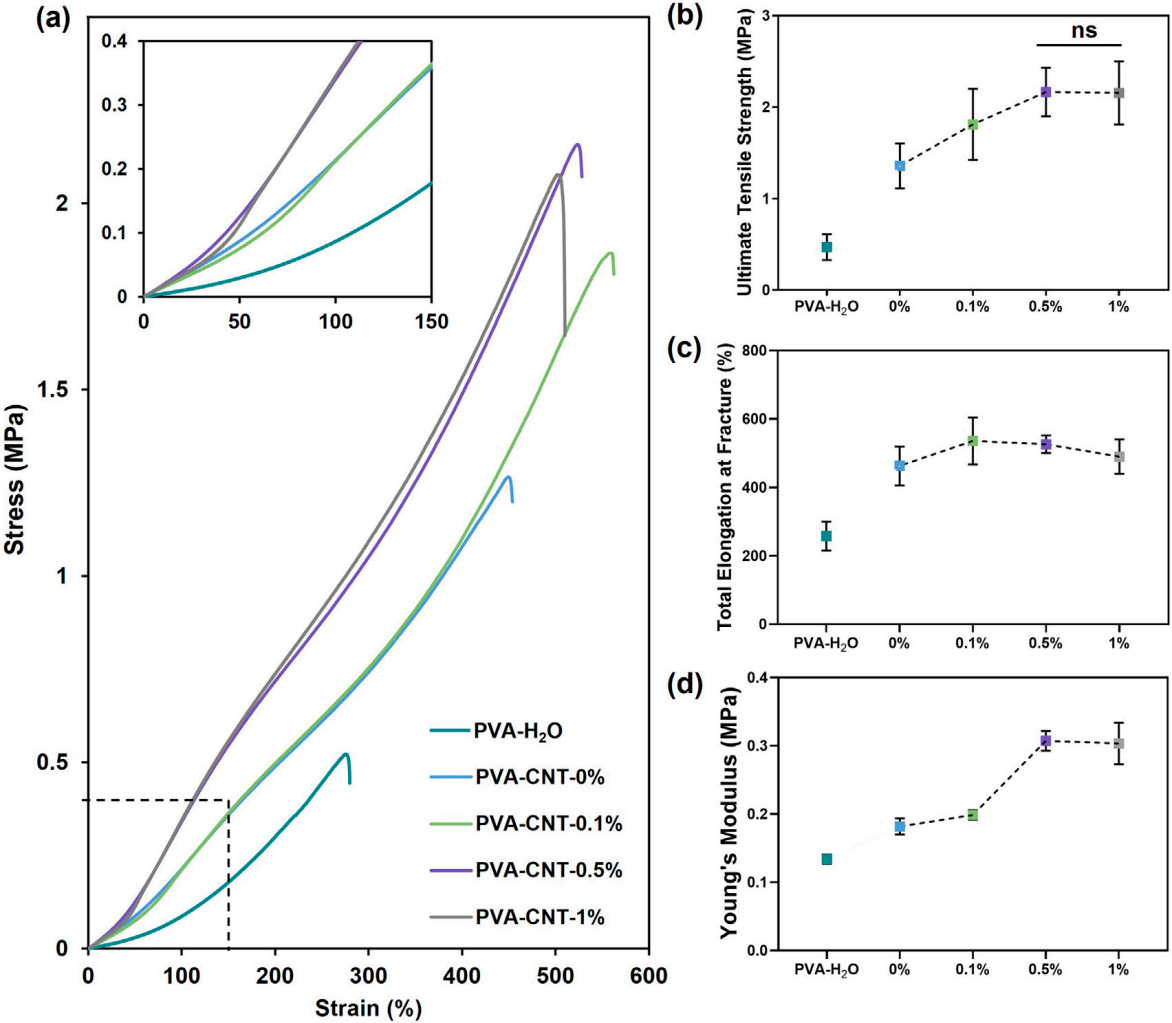
The mechanical properties of PVA-cryogel nanocomposites. **(a)** Engineering stress-strain curves, **(b)** relationship between CNT content and ultimate tensile strength, **(c)** total elongation at fracture, and **(d)** Young’s modulus. Statistical significance of differences between groups was determined by analysis of variance (ANOVA) with Fisher’s parametric test for *post hoc* comparisons. Data are presented as mean ± SD (n = 7–8).

In our study, PVA cryogels prepared from H_2_O solutions exhibited significantly lower mechanical strength compared to samples synthesized using a DMSO/H_2_O mixture (0.47 ± 0.14 MPa vs 1.36 ± 0.25 MPa, respectively; p < 0.05) (Figure 3). This difference may be attributed to the more homogeneous structure of the DMSO/H_2_O hydrogels, which contain a greater number of crystalline domains (Xu et al., 2020) formed after a single freeze-thaw cycle, as well as to the replacement of DMSO, a thermodynamically good solvent for PVA, with water, a poor solvent.

An investigation into the effect of nanofillers on the mechanical properties of the polymer hydrogel matrix revealed a gradual increase in tensile strength with increasing CNT concentration from 0% to 0.5%. The mechanical strength of the polymer nanocomposite cryogel containing 0.5% CNT was 59% higher than that of the pure polymer matrix, reaching 2.17 ± 0.26 MPa. However, further increasing the concentration of carbon nanoparticles beyond 0.5% did not result in a statistically significant increase in mechanical strength (p = 0.95). In particular, the mechanical strength of the cryogel sample with 1% CNT was measured at 2.16 ± 0.34 MPa (Figures 3a,b).

The beneficial effect of incorporating CNTs at concentrations ranging from 0% to 0.5% is attributed to the high strength of the nanofiller (Abubakre et al., 2023) and its interaction with the polymer matrix (Heidarshenas et al., 2019). These interactions restrict the mobility of PVA chains, leading to stabilization of the amorphous regions and, consequently, enhanced mechanical performance of the composite material (Heidarshenas et al., 2019). Moreover, the incorporation of CNTs into PVA-based nanocomposites has been reported to increase the overall crystallinity of the polymer while simultaneously reducing the size of crystalline domains, which also contributes to improved mechanical strength (Xu et al., 2020; Naebe et al., 2008). Although some studies emphasize the need for CNT functionalization to improve interfacial compatibility, in our system, the transition from DMSO to water likely facilitated the formation of strong interactions between the polymer and the nanofiller. These interactions are partially governed by hydrogen bonding between oxygen-containing groups on CNT surfaces and PVA chains, as well as van der Waals forces. As a result, CNTs help prevent crack propagation and dissipate mechanical energy by effectively interacting with the polymer matrix (Salah et al., 2019). However, as CNT content increases, the distance between nanoparticles decreases, leading to aggregation and a reduction in the reinforcing effect of the filler (Górska et al., 2021). A similar tendency was reported by Tong et al. in their synthesis of PVA-based cryogels containing oxidized CNTs (Tong et al., 2007), and by Pykin et al. for CNTs modified with PVA (Pykin et al., 2025). For such nanocomposites, the maximum strength typically reaches around 2 MPa. Due to their high water content (usually ∼80–90 wt.%), hydrogels exhibit a critical strength limit that supports tissue-like properties but restricts mechanical performance. Reducing water content below ∼60–70% can improve strength but often compromises biocompatibility (Means and Grunlan, 2019). In our case, we achieved a significant improvement in material strength while maintaining a high water content and avoiding the need for chemical modification. As noted earlier, amorphous chains associated with water through hydrogen bonds maintain part of the PVA in its amorphous state, preventing complete polymer crystallization and thus giving it a rubber-like texture (Masri et al., 2017). The PVA cryogel’s high dimensional stability and properties comparable to biological tissues were provided by the crystalline domains of PVA molecules and a high-water content. The total elongation at fracture of cryogels prepared based on the DMSO/H_2_O mixture amounted to 462.8% ± 56.8%, almost twice the value obtained for water cryogels (p < 0.0001) (Figures 3a,c). A notable increase in relative elongation to 535.9% ± 68.8% (p < 0.05) was observed upon the addition of 0.1% CNT to the polymer matrix. However, cryogel samples containing 0.5% and 1% CNT did not demonstrate statistical significance in comparison to the low-filled nanocomposite (Figures 3a,c).

The Young’s modulus, characterizing material stiffness within physiological load ranges, varied from 0.18 ± 0.01 MPa to 0.31 ± 0.01 MPa for cryogel samples containing 0.1% and 0.5% nanofiller, respectively (p < 0.0001) (Figures 3a,d). A further increase in CNT concentration did not result in significant changes in stiffness (p = 0.68). Comparing the stiffness of the studied materials with human body tissues, an advantage of cryogels over elastic polymers was identified, which often exceed physiological values of the Young’s modulus by several times (Oveissi et al., 2019). Based on the results of the uniaxial tension, the optimal CNT content in the PVA nanocomposite cryogel composition was determined to be 0.5%.

Although the developed nanocomposite hydrogels still exhibit significantly lower tensile strength compared to the clinically used and commercially available ePTFE (up to 30 MPa) (Rezvova et al., 2024), their mechanical properties closely approach, and in some cases even exceed, those of the native human aortic valve (1.74 MPa) (Kalejs et al., 2009). The reduced stiffness of the PVA-CNT composites relative to ePTFE (∼11 MPa) may be advantageous, as excessively rigid materials have been shown to cause mechanical damage to blood cells and disrupt physiological blood flow patterns (Rezvova et al., 2023).

#### 3.3.2 Elastic hysteresis

**FIGURE 4:**
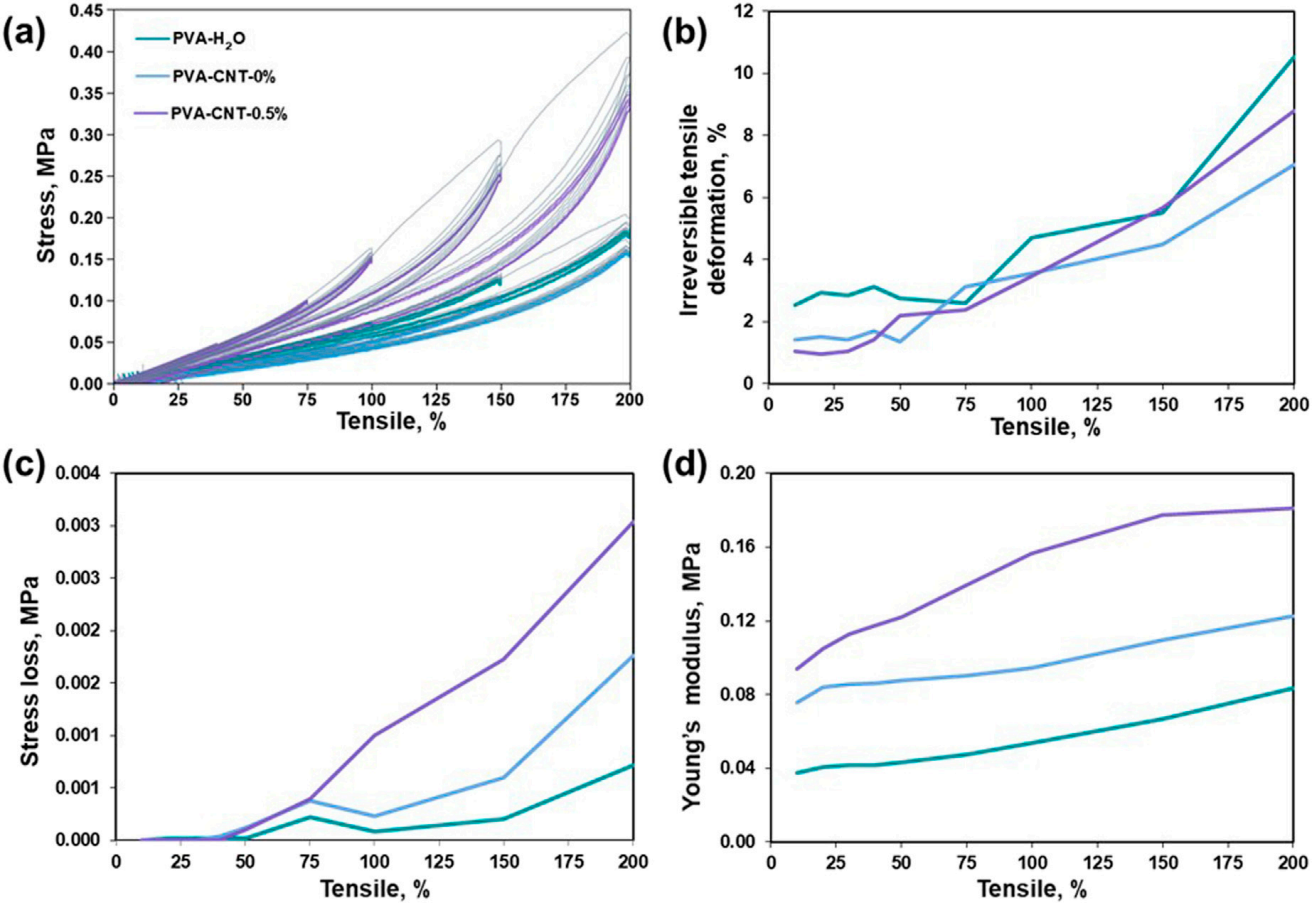
Research results on elastic hysteresis. **(a)** Stress-strain hysteresis loops, **(b)** irreversible deformation during cyclic loading, **(c)** stress‐softening during cyclic loading, **(d)** alterations in Young’s modulus during cyclic loading.

The Mullins effect, a phenomenon characterized by stress softening under cyclic loading conditions and initially observed in filled elastomers, plays a pivotal role in predicting both the short‐ and long‐term mechanical behaviour of polymer composites under repeated loading conditions (Diani et al., 2009).The stress–strain responses of PVA nanocomposites during subsequent loading loops indicate that the initial loading cycle induces microstructural rearrangements that irreversibly alter the material’s stress–strain behaviour (Figure 4a).

As the magnitude of cyclic elongation increases, irreversible deformation becomes more pronounced in all tested samples (Figure 4b). It is noteworthy that, even at cyclic elongations of 10%, none of the cryogels examined exhibit complete elastic recovery. Concurrently, when the cryogel samples are subjected to cyclic elongations of up to 50% (Figure 4c), no stress loss is observed, indicating the absence of structural deterioration in the molecular crosslinking of the composite within the investigated loading range. However, beyond a cyclic tensile strain of 50%, we observed a phenomenon in which repeated stretching of the material to a constant magnitude leads to a reduction in the stress required to maintain the given elongation (Lakes, 2009). This aligns with the theory of stress redistribution in a polymer matrix with nanoparticles, which form strong interfacial bonds that facilitate efficient transmission and distribution of mechanical stresses (Coleman et al., 2006; Chou et al., 2010). In elastomers, this effect reflects the rearrangement of the polymer network following the initial deformation. Further elongation of the cryogels leads to the accumulation of irreversible strain and a noticeable decrease in the force required to achieve it. Notably, PVA-CNT exhibits nonlinear stiffening with increasing length (Figure 4d), distinguishing it from PVA-H_2_O and PVA-mix, which show a linear increase in Young’s modulus.

### 3.4 Cytotoxicity of cryogel nanocomposites

The interaction between cell and hydrogel is complex and dynamic, which exerts significant impacts on tissue physiological (e.g., cell spreading, proliferation, migration, differentiation, *etc.*) and pathological processes, such as cell apoptosis, fibrosis, immunological rejection, *etc.* (Cao et al., 2021).

**FIGURE 5:**
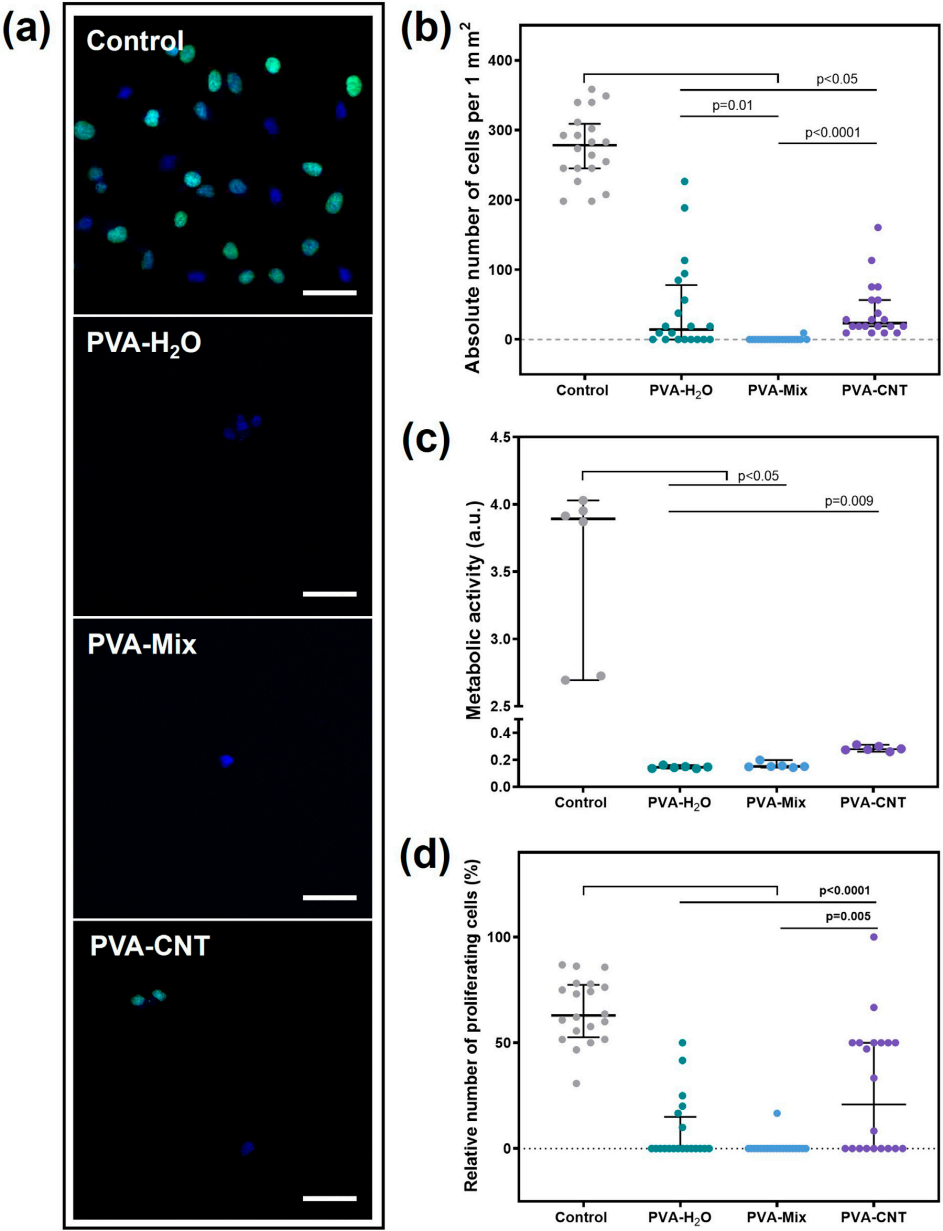
Results of the cytotoxicity study of PVA-based nanocomposite cryogels using the Ea.hy 926 cell line. **(a)** Fluorescent microscopic images of films after cell cultivation (proliferation), magnification ×200, **(b)** absolute number of cells adhered to the material surfaces, **(c)** metabolic activity of cells, and **(d)** proliferative activity of cells. Scale bars: 50 μm. Statistical significance of differences between groups was assessed using the Kruskal–Wallis test followed by Dunn’s *post hoc* correction for multiple comparisons. Data are presented as median with interquartile range (Me [25%; 75%]).

A distinctive feature of the PVA-based cryogels in this study was a significant reduction in their adhesion capacity to cells compared to the culture plastic (p < 0.05) (Figures 5a,b). The lowest number of cells was observed on the surface of the cryogel prepared using a DMSO/H_2_O mixture, with 0.00 [0.00; 0.00] cells/mm^2^. The low adhesion capability of PVA is due to the absence of cellular adhesion sites, which can be represented by amino groups, ester groups, and other functional groups (Cao et al., 2021; Ino et al., 2013). However, the introduction of CNTs into the structure of the polymer cryogel led to an increase in the number of cells adhered to the surface, up to 23.6 [18.87; 56.60] cells/mm^2^, which was still 10 times lower than control values (Figures 5a,b). The observed heterogeneity in cell distribution on PVA-H_2_O materials may be related to the surface topography. Aqueous cryogels can form regions with a denser polymer network and relatively large, irregularly shaped pores up to 1–2 µm in size, as confirmed by SEM analysis. In the case of nanocomposites, surface irregularity also affects cell distribution; in addition, carbon nanotubes may create localized areas of higher concentration, promoting enhanced cell adhesion compared to the PVA-mix sample without nanofillers. SEM analysis of the latter revealed a more uniform pore structure with significantly smaller pores.

The metabolic and proliferative activities of Ea.hy 926 cells correlated with the total number of cells and were statistically significantly higher for culture plastic (Figures 5a,c,d). Nevertheless, the observed result is a consequence of the low adhesion capability, not the toxicity of the material. The number of proliferating cells on the nanocomposite surface was significantly higher compared to cryogels without the carbon nanofiller, with 20.83 [0.00; 50.00] % and 0.00 [0.00; 0.00] %, respectively (p = 0.05) (Figures 5a,d). Thus, the addition of CNT to the polymer matrix not only did not increase the toxicity of the materials but also improved cell adhesion and proliferation. This is likely due to the emergence of nano-sized fibers on the polymer surface, which may have been attractive for the Ea.hy 926 cell culture (Rezvova et al., 2022). Nevertheless, the target application area for nanocomposites does not require endothelialisation of the polymer surface. The primary condition for the material to function effectively in the bloodstream is thromboresistance. It should also be noted that the use of a DMSO/H_2_O mixture as a solvent did not affect the toxicity of polymer hydrogel matrices.

### 3.5 Hemocompatibility assessment of cryogel nanocomposites *in vitro*

The first event that occurs when a foreign material comes into contact with blood is the adsorption of protein molecules. This is because the diffusion coefficient of proteins in plasma is significantly higher than that of the smallest formed blood elements–platelets (10^−6^–10^–7^ cm^2^/s and 10^–9^ cm^2^/s, respectively) (Weber et al., 2018). The nature of protein adsorption depends on several factors related to the adsorbing surface, including hydrophobicity/hydrophilicity, electrostatic charge, chemical reactivity, surface topography, molecular mobility, and the degree of amorphousness or crystallinity (Rahmati and Mozafari, 2018). Hydrophobic surfaces with high roughness are particularly prone to non-specific protein adsorption (Rahmati and Mozafari, 2018).

**FIGURE 6:**
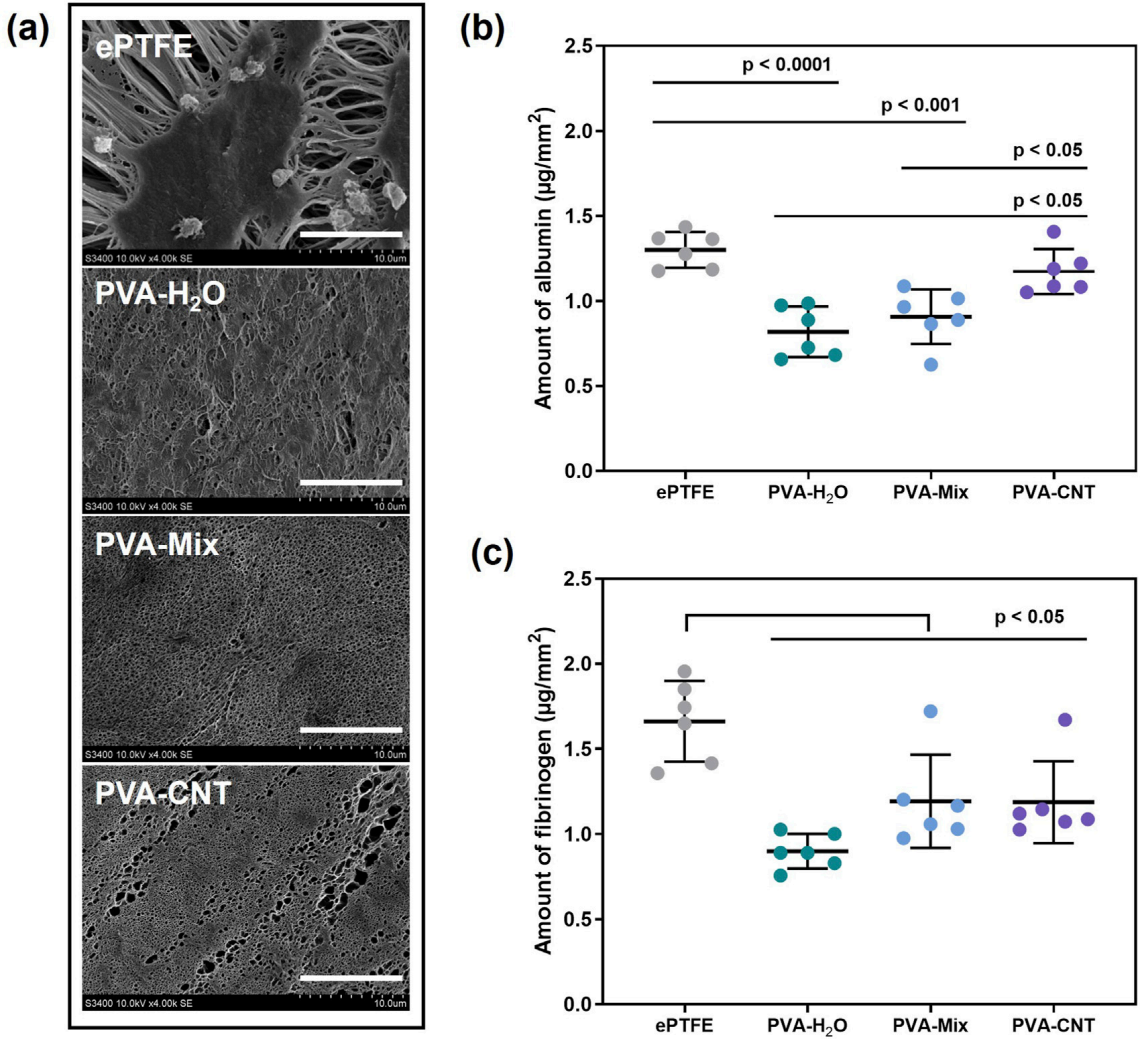
Results of the *in vitro* hemocompatibility test. **(a)** SEM images obtained after exposing the biomaterial samples to platelet-rich plasma, scale bars: 10 μm, **(b,c)** amounts of protein adsorbed on the surface of the studied polymers: fibrinogen and albumin. Statistical significance of differences between groups was determined by analysis of variance (ANOVA) with Fisher’s parametric test for *post hoc* comparisons. Data are presented as mean ± SD (n = 6).

The current study found that cryogels exhibited significantly lower affinity for protein adsorption compared to the ePTFE material (p < 0.05). This difference is likely due to the higher hydrophobicity and greater porosity of ePTFE compared to cryogels (Figure 6). Our water contact angle measurements confirmed the high hydrophilicity of the cryogels, which may further explain their lower protein adsorption. In contrast, ePTFE is known to exhibit a water contact angle of up to 110° (Rezvova et al., 2024; Rahmati and Mozafari, 2018), reflecting its strongly hydrophobic nature.

Overall, a greater amount of fibrinogen was observed on the surface of polymer films compared to albumin (Figures 6b,c). Numerous studies have examined the influence of hydroxyl (-OH) groups on protein adsorption. It has been demonstrated that water can effectively compete with fibrinogen for adsorption on surfaces containing hydroxyl groups. This finding supports the observation that fibrinogen interacts more strongly with the cryogel surface than albumin (Rahmati and Mozafari, 2018). Our experiments also showed a slight increase in albumin adhesion on the surface of the nanocomposite cryogels compared to the pure PVA cryogels. This may be attributed to subtle changes in surface microstructure and a tendency toward increased hydrophobicity upon the incorporation of nanoparticles into the polymer network.

In addition to plasma proteins, implants made from synthetic polymeric materials also come into contact with formed blood elements, such as erythrocytes. The evaluation of hemolysis levels reflects the ability of the tested biomaterials to induce erythrocyte lysis (Weber et al., 2018). The lowest hemolysis was observed in the cryogel groups, with values of 0.01 [0.01; 0.02] %, which was significantly lower than that of the ePTFE group (p < 0.05) (Table 3). However, none of the materials exceeded the permissible hemolysis threshold of 2%, indicating low cytotoxicity of the studied polymers toward erythrocytes. Previous studies on PVA-silver nanocomposites have shown that both the polymer-to-nanofiller ratio and the number of freeze-thaw cycles can influence material toxicity, likely due to increased network density and reduced water content, which may impair biocompatibility (Chaturvedi et al., 2014). Our results demonstrate that the selected composition enables the development of a safe material, with no signs of increased erythrocyte toxicity.

**TABLE 3:** Hemolysis results for polymeric samples.

Materials	Hemolysis degree, %				
	Min	25%	Median	75%	Max
ePTFE	0.10	0.12	0.12	0.13	0.15
Cryogels based on polyvinyl alcohol (PVA)					
PVA-H_2_O	0.01	0.01	0.01	0.02	0.02
PVA-Mix	0.01	0.01	0.01	0.02	0.02
PVA-CNT	0.01	0.01	0.01	0.02	0.03

Another group of blood elements, the damage of which during the implantation of artificial replacements can lead to serious complications, are platelets. Despite the physiological geometry of modern cardiovascular implants, such as polymeric heart valve prostheses and compliant vascular grafts, the chemical nature of the blood-contacting surface can significantly influence platelet adhesion, activation, and the risk of thrombosis (Rezvova et al., 2023; Li et al., 2014). Hydrophobic polymeric materials containing -CH_2_-CH_3_, aromatic groups, or fluorine groups (-C_2_F_4_-) typically promote thrombus formation, partly due to the early adsorption of protein molecules. In contrast, hydrophilic functional groups, especially hydroxyl (-OH) groups, reduce platelet adhesion and activation (Long et al., 2016). After contact with platelet-rich plasma, no formed elements were detected on the surface of the polymeric cryogels, which is most likely associated with the high hydrophilicity of the material surfaces, as confirmed by the water contact angle measurements (Section 3.2.5). This observation correlates with the results of experiments involving cells and protein molecules, as well as with literature data (Figure 6) (Cutiongco et al., 2016). In contrast, 4,238 [1,156; 23,503] platelets/mm^2^ were found on the surface of the ePTFE polymer. These platelets showed varying degrees of deformation, indicating activation of the cells. Predominantly, type III-IV platelets were observed, with fewer type V platelets and occasional type II platelets. Thus, cryogels exhibit lower thrombogenicity compared to clinically used ePTFE.

Upon contact with an adsorbed protein layer or the free surface of a foreign material, platelets may be repelled; however, this does not exclude their activation and the initiation of aggregation processes. Therefore, in this study, the degree of platelet aggregation in plasma that had been in contact with the experimental samples was separately assessed. The maximum platelet aggregation observed for the studied materials is presented in Table 4. No statistically significant differences were detected between the groups for this parameter.

**TABLE 4:** Maximum platelet aggregation for the samples of polymeric materials studied.

Materials	Platelet aggregation, %				
	Min	25%	Median	75%	Max
Intact plasma	83.16	84.12	85.92	89.10	89.85
ePTFE	87.80	88.02	89.12	89.71	90.33
Cryogels based on polyvinyl alcohol (PVA)					
PVA-H_2_O	82.35	84.76	88.21	90.48	93.13
PVA-Mix	87.90	88.22	88.62	89.47	91.23
PVA-CNT	89.30	89.98	90.61	91.19	91.35

PVA cryogels and other synthetic hydrogels have demonstrated high hemocompatibility. Numerous studies have focused on the development of small-diameter vascular grafts based on such macromolecular matrices, where low thrombogenicity is critically important (Jeong et al., 2021). However, the thrombogenic potential of carbon nanotubes (CNTs) remains a challenge for various biomedical applications (Fedel, 2020). CNTs can adsorb blood plasma proteins, particularly albumin and fibrinogen, induce erythrocyte lysis, and activate and aggregate platelets (Fedel, 2020; Semberova et al., 2009). Nevertheless, it has been shown that surface modification of CNTs can reduce their thrombogenic potential (Fedel, 2020).

The high hemocompatibility of the nanocomposite cryogels investigated in this study is attributed to the fact that the nanofiller is fully embedded within the polymer matrix, as confirmed by SEM analysis. It is most likely that the carbon nanotubes are encapsulated by polyvinyl alcohol molecules (Hajeeassa et al., 2018). Although a tendency toward increased albumin adsorption on the nanocomposite surface was observed in tests using a concentrated protein solution, this did not promote platelet adhesion or aggregation in experiments using platelet-rich plasma, which naturally also contains albumin. This effect may be associated with the unique properties of albumin, which make it a suitable thromboresistant coating for various synthetic polymers (Li et al., 2014). On the other hand, under our experimental conditions, only a small amount of protein may adsorb from plasma due to the relatively low concentration and small volume used. This may not produce a detectable effect at this stage but could become more pronounced over time as adsorption accumulates. Therefore, long-term *in vitro* or *in vivo* studies under flow conditions are required to confirm these findings.

Considering the hemocompatibility test results comprehensively, it can be concluded that CNTs do not adversely affect the properties of PVA-based polymer cryogels. Moreover, the cryogels developed in this study demonstrate more favorable properties for use in cardiovascular surgery compared to ePTFE materials.

### 3.6 Biocompatibility assessment of cryogel nanocomposites *in vivo*

**FIGURE 7:**
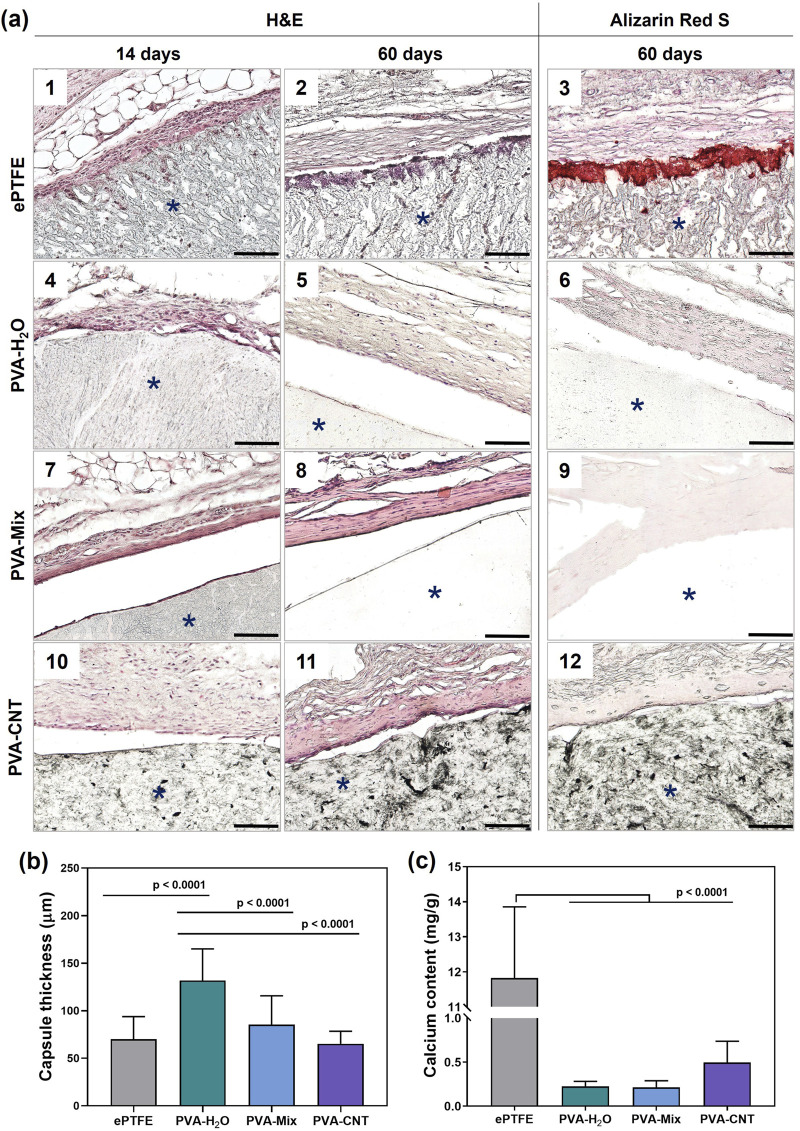
Histological analysis of implanted polymeric materials subcutaneously in rats. **(a)** Cross-sections stained with H&E of explanted materials with surrounding tissue at 14-day (1, 4, 7, 10), and 60-day (2, 5, 8, 11) time points, and stained with Alizarin red S at 60-day (3, 6, 9, 12) time point, **(b)** quantification of collagen-based connective tissue capsule thickness at 60 days, and **(c)** calcium content in the explanted samples. Asterisk (*) denotes material implants. Scale bars: 100 μm.

The biocompatibility of the obtained materials was assessed through microscopic analysis of the tissue morphology surrounding the implants. Two weeks post-implantation, the observed tissue response corresponded to a typical inflammatory reaction to surgical trauma (Shakya et al., 2013). Moderate macrophage infiltration was noted, along with the presence of foreign body giant cells (FBGCs) in both investigated materials–ePTFE and PVA-H_2_O (Figure 7a). It is well established that implants with a higher surface-to-volume ratio, such as porous materials, induce greater macrophage and FBGC recruitment compared to smooth-surfaced implants, aligning with our observations (Sheikh et al., 2015). Additionally, biomaterial surface chemistry plays a crucial role in modulating the foreign body response (Sheikh et al., 2015), with hydrophilic polymeric materials or coatings generally reducing inflammation (Sutthiwanjampa et al., 2023).

Although ePTFE, which contains fluorine groups, and CNT-filled cryogels differ in chemical composition, the latter did not elicit a significantly different inflammatory response compared to single-component cryogels. Cellular infiltration was observed on the surface of ePTFE, likely due to its porous and hydrophobic nature (Figure 7a). In contrast, polymeric cryogels lacked cellular elements within their structure, and native tissues surrounding these implants showed no adhesion to the cryogel surface, detaching during histological sample preparation.

A fibrous collagen capsule was observed around all samples as early as 14 days post-implantation (Figure 7a), with fatty infiltration associated with collagen fibrils detected in the ePTFE sample (Figure 7). All samples exhibited comparable levels of neovascularization, with 1-3 newly formed blood vessels per field of view at ×400 magnification.

Local inflammation decreased with the formation of connective tissue capsules of moderate thickness around both the cryogels and ePTFE 2 months after implantation (Figures 7a,b). The average capsule thicknesses were as follows: 70 ± 24 µm for ePTFE, 132 ± 33 µm for PVA-H_2_O, 85 ± 30 µm for PVA-mix, and 65 ± 13 µm for PVA-CNT (Figures 7a,c).

Typically, fibrosis forms around biomaterials or implants as a result of the foreign body reaction at the tissue–implant interface. This process isolates the implant and the associated inflammatory response from the surrounding tissue environment, serving as an indicator of the material’s biocompatibility. Among the studied samples, the thickest connective tissue capsule was observed around the PVA-H_2_O implants (p < 0.0001). Interestingly, despite this observation, literature data report the low immunogenicity, non-toxicity, and high biocompatibility of PVA hydrogels (Barbon et al., 2020).

A dense network of neovascularization was noted in the ePTFE samples, likely associated with progressing calcification accompanied by cellular infiltration of the polymeric matrix. In contrast, no cells were detected within the hydrogel matrices, even at later stages of implantation. After 60 days, a reduced cell density was observed around the implant perimeters, indicating a decline in inflammation (Figure 7a). The predominant cell type was active fibroblasts with characteristic spindle-shaped morphology, as identified by H&E staining. Furthermore, no signs of material degradation or abnormal histopathological changes in the surrounding subcutaneous tissue were observed in any of the studied samples (Figure 7a).

**FIGURE 8:**
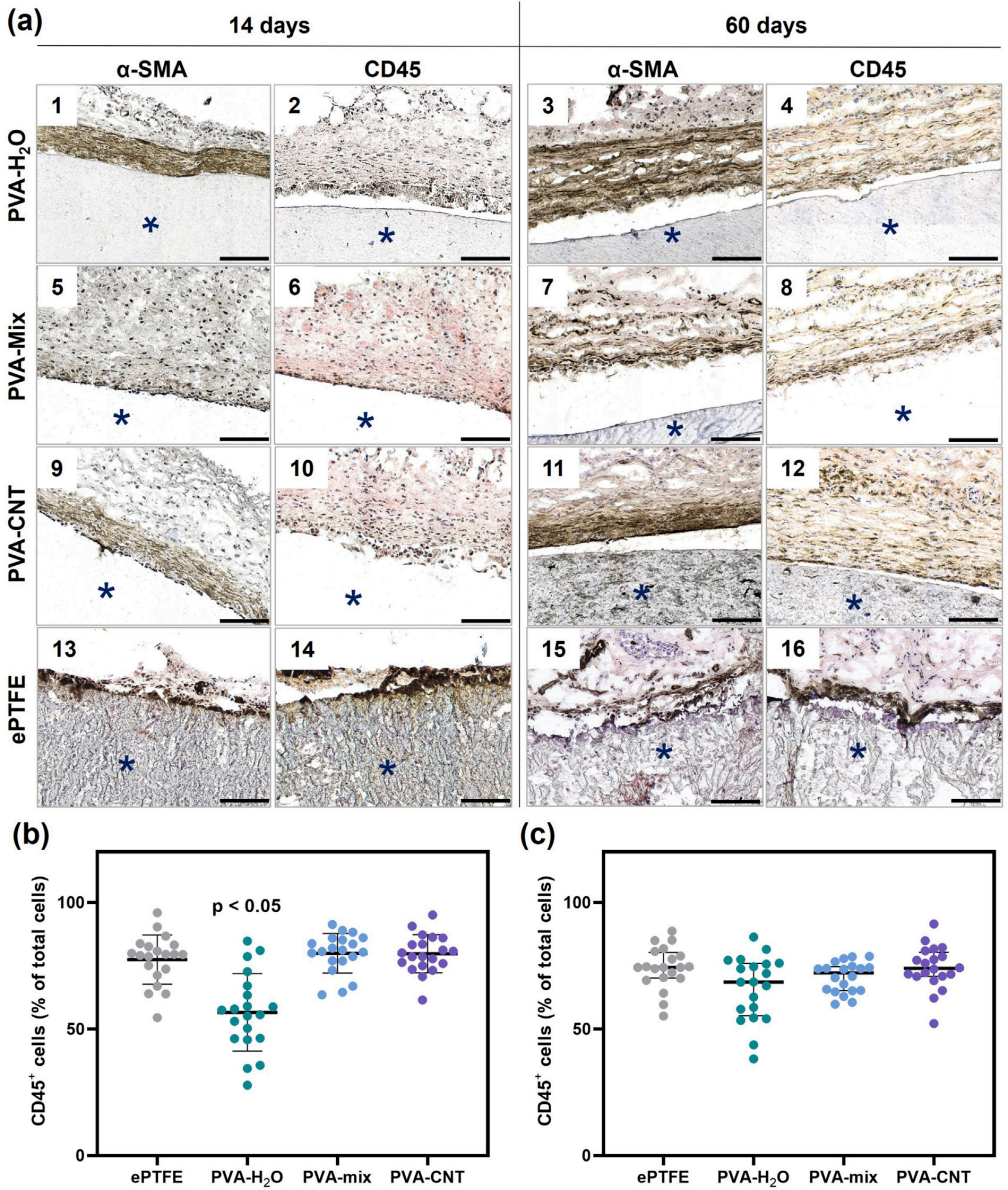
Immunohistochemistry analysis of implanted polymeric materials subcutaneously in rats. **(a)** Cross-sections immunostained (brown) with an antibody to CD45 (even numbers) and α-SMA (odd numbers) at 14 and 60 days. The asterisk (*) denotes material implants. Scale bars: 100 μm. **(b)** and **(c)** semi-quantitative assessment of the relative number of immune cells (CD45^+^) in the peri-implant area at 14- and 60-day post-implantation. Statistical significance of differences between groups was assessed using the Kruskal–Wallis test followed by Dunn’s *post hoc* correction for multiple comparisons. Data are presented as median with interquartile range (Me [25%; 75%]).

In addition to the H&E analysis of inflammation, we performed anti-CD45 staining to assess leukocyte infiltration in the tissue surrounding the implants and α-SMA staining to identify collagen-producing myofibroblasts (Figure 8). Leukocytes were detected at both control time points during the tissue regeneration process around the implants (Figure 8a). Notably, after 60 days, an increased number of myofibroblasts was observed within the fibrous capsule surrounding the implants, indicating an active process of connective tissue formation. Conversely, the overall number of leukocytes in the surrounding dermis significantly decreased over the course of implantation. At each time point, a higher cell density was observed around the perimeter of the implanted ePTFE matrices compared to other materials (Fig. A-14,16). Semi-quantitative analysis of CD45-positive cells revealed an advantage of the PVA-H_2_O material after 14 days of implantation, with the lowest percentage of leukocytes relative to the total number of cells in the peri-implant tissue (p < 0.05). By day 60, this difference was no longer evident, as the relative number of leukocytes decreased for all materials to 69%–74%, with no statistically significant differences between the groups (p > 0.05) (Figures 8b,c).

Therefore, no significant differences were observed in the immune response elicited by the materials, suggesting that the incorporation of nanoparticles into the matrix structure does not compromise its biocompatibility in the *in vivo* setting. However, macrophage M1/M2 polarization plays a key role in modulating the immune response to biomaterials. Distinguishing between these subtypes provides deeper insight into the nature and resolution of inflammation. Thus, future studies should investigate macrophage phenotypes to more comprehensively evaluate the biocompatibility of the materials.

Synthetic materials, like biological ones, can exhibit a tendency toward calcification, which is often associated with the inflammatory response surrounding the implant (Lee et al., 2021). After 60 days of subcutaneous implantation in rats, the calcium content in the ePTFE samples was 11.72 mg/g of dry tissue – 27 times higher than that observed in the nanocomposite PVA cryogels (p < 0.0001) (Figure 7c). Analysis of calcification patterns in the tissue samples revealed that, in ePTFE implants, calcifications predominantly formed at the interface between the material and surrounding tissues (Figure 7a), while no calcium deposits were detected in the nanocomposite cryogels under microscopic examination. This result may be attributed to differences in the hydrophobicity and porosity of the materials. It is well established that more hydrophobic materials tend to elicit a stronger inflammatory response, particularly in the case of highly hydrophobic ePTFE, which contains C–F bonds in its structure (Shan et al., 2022). Porosity affects cell infiltration, vascularization, and consequently, the degree of inflammation (Luthra et al., 2020). In the context of designing blood-contacting devices, it is important to note that interactions between blood components and biomaterials can initiate a cascade of events, starting with protein adsorption and potentially leading to calcification. In this regard, the high affinity of ePTFE for protein and platelet adhesion represents an additional complicating factor (Lejay et al., 2023). In contrast, the hydrophilic nature of PVA cryogels results in minimal adhesion of proteins, platelets, and cells. This intrinsic non-adhesive property is expected to contribute to their long-term resistance to calcification in blood-contacting applications.

## 4 Conclusion

Based on the results of this study, we conclude that it is feasible to produce PVA nanocomposites through the cryostructuring of polymer solutions containing pre-dispersed carbon nanotubes (CNTs) in a DMSO/H_2_O solvent mixture. The incorporation of CNTs at concentrations up to 0.5 wt.% by polymer weight enhanced the thermal stability and mechanical strength of the nanocomposites by 59% compared to single component cryogels. The reinforcement effect of CNT-loaded cryogels is evidenced by an increased Young’s modulus and pronounced strain hardening at large deformations. Despite an increase in the adhesive properties of the PVA-CNT hydrogels toward Ea.hy 926 cells, the number of cells adhering to the cryogel surfaces remained significantly lower than on control culture plastic. Therefore, the materials can be considered to exhibit anti-adhesive behavior. The nanocomposite cryogels demonstrated high hemocompatibility, as confirmed by blood component tests. Minimal adsorption of albumin and fibrinogen was observed, with no platelet adhesion detected upon exposure to platelet-rich plasma. Additionally, platelet aggregation levels remained comparable to those of untreated plasma. The materials exhibited a minimal inflammatory response and no signs of calcification, in contrast to the clinically used ePTFE, further supporting their biocompatibility. The presence of CNTs did not trigger an atypical foreign body response, and SEM analysis confirmed the homogeneous distribution of CNTs encapsulated within the polymer matrix.

Overall, PVA-based nanocomposite cryogels produced *via* cryostructuring with dispersed CNTs represent a promising material for the development of cardiovascular implants and related biomedical applications. However, additional studies are needed to confirm the safety of these materials for use in heart valve or vascular graft applications. In particular, cell-based assays and flow-dependent hemocompatibility tests should be performed to assess the material’s ability to support cell adhesion and the survival of blood components under physiologically relevant conditions. Furthermore, future studies should explore their antibacterial potential and electrical conductivity for tissue engineering applications, alongside strategies to achieve nanoparticle alignment within the matrix, such as mechanical loading or external magnetic fields, to further enhance mechanical properties and introduce anisotropy.

## Data Availability

The authors confirm that the data supporting the findings of this study are available within the article. The data related to elastic hysteresis analyzed in this study are available at https://doi.org/10.17632/7f2jb5tfh8.1. The software developed for data analysis is accessible at https://github.com/onisps/hysteresis_analisys.
